# Genome-wide association studies of severe *P. falciparum* malaria susceptibility: progress, pitfalls and prospects

**DOI:** 10.1186/s12920-019-0564-x

**Published:** 2019-08-14

**Authors:** Delesa Damena, Awany Denis, Lemu Golassa, Emile R. Chimusa

**Affiliations:** 1 Division of Human Genetics, Department of Pathology, Institute of Infectious Disease and Molecular Medicine, Faculty of Health Sciences, University of Cape Town, Private Bag, Rondebosch, Cape Town, 7700 South Africa; 2 Aklilu Lema Institute of Pathobiology, Addis Ababa University, PO box 1176, Addis Ababa, Ethiopia

**Keywords:** Genome-wide association study, *P. falciparum* malaria, Susceptibility, Resistance, Heritability, Pathways, Fine-mapping, Multi-omics, Systems biology

## Abstract

**Background:**

*P. falciparum* malaria has been recognized as one of the prominent evolutionary selective forces of human genome that led to the emergence of multiple host protective alleles. A comprehensive understanding of the genetic bases of severe malaria susceptibility and resistance can potentially pave ways to the development of new therapeutics and vaccines. Genome-wide association studies (GWASs) have recently been implemented in malaria endemic areas and identified a number of novel association genetic variants. However, there are several open questions around heritability, epistatic interactions, genetic correlations and associated molecular pathways among others. Here, we assess the progress and pitfalls of severe malaria susceptibility GWASs and discuss the biology of the novel variants.

**Results:**

We obtained all severe malaria susceptibility GWASs published thus far and accessed GWAS dataset of Gambian populations from European Phenome Genome Archive (EGA) through the MalariaGen consortium standard data access protocols. We noticed that, while some of the well-known variants including *HbS* and *ABO* blood group were replicated across endemic populations, only few novel variants were convincingly identified and their biological functions remain to be understood. We estimated SNP-heritability of severe malaria at 20.1% in Gambian populations and showed how advanced statistical genetic analytic methods can potentially be implemented in malaria susceptibility studies to provide useful functional insights.

**Conclusions:**

The ultimate goal of malaria susceptibility study is to discover a novel causal biological pathway that provide protections against severe malaria; a fundamental step towards translational medicine such as development of vaccine and new therapeutics. Beyond singe locus analysis, the future direction of malaria susceptibility requires a paradigm shift from single -omics to multi-stage and multi-dimensional integrative functional studies that combines multiple data types from the human host, the parasite, the mosquitoes and the environment. The current biotechnological and statistical advances may eventually lead to the feasibility of systems biology studies and revolutionize malaria research.

## Background

*Plasmodium falciparum*, the causative agent of severe malaria, has been infecting humans for at least 5000-10,000 years following the advent and expansions of agriculture [1–3]. Malaria still poses a huge social, economic and health problems in several low-income countries, particularly in sub Saharan Africa [4, 5]. *P. falciparum* infects millions and kills hundreds of thousands of African children each year. However, this constitutes only a small proportion (1%) of the populations in endemic areas in which the infections progress to severe malaria such as profound anemia or cerebral malaria [6, 7].

Comprehensive understanding of the genetic basis of resistance and susceptibility to severe malaria is crucial to understand the molecular mechanisms of host-parasite interactions that can inform the development of effective therapeutics, vaccination, diagnostics and risk prediction strategies [8, 9]. To this end, GWASs have recently been implemented in malaria endemic areas and replicated some of the well-known variants including *HbS* and *ABO* blood group [10–13]. Despite the fact that malaria is expected to drive several protective alleles to high frequencies that can be captured by GWAS approach, it is unclear why only limited number of novel variants were identified of which a small fraction was replicated across endemic populations. Some of the contributing factors for this discrepancy might include small sample sizes, the genetic diversity of the malaria endemic populations and allelic heterogeneity of malaria protective alleles among others. On the other hand, several association signals distributed across the genome that didn’t pass GWAS significance threshold were observed in these studies [10–13]; suggesting the possible existence of polygenic effects. This raises several key questions including 1) What is the genetic architecture of malaria susceptibility/resistance? 2) What is the heritability of malaria susceptibility and its distribution across the genome? and 3) What is the extent and pattern of epistasis and pleiotropy at genome wide scale?

Here we review the current status of malaria susceptibility GWASs and provide guidance to future research directions. We begin by assessing the progress and pitfalls of severe malaria susceptibility GWASs and discuss the biology of the novel variants. We then provide an overview of the recent progresses in post-GWAS approaches and discuss how these methods can be implemented in severe malaria susceptibility studies to better understand the underlying biology. We conclude by discussing on research areas where further works are needed in light of the global malaria eradication efforts.

## Results

### Severe *P. falciparum* malaria susceptibility GWASs: progress and pitfalls

In malaria endemic areas where repeated *P. falciparum* infection is very common, the majority of children recover from malaria. However, a small proportion of infections progress to the severe form of the disease such as severe anaemia, cerebral malaria, acidosis and respiratory distress [7]. Cerebral malaria is the commonest cause of death characterized by rapid onset of generalized convulsion followed by coma (a Blantyre coma score of less than 3 in the presence of *P. falciparum* parasitaemia). Severe anaemia is defined as a haematocrit of < 15% or haemoglobin < 5 g/dl in the presence of *P. falciparum* parasitaemia [14]. Although the clinical outcome of malaria is determined by several factors including infection rate, parasite genetics and the environment, the host-genetics factor contribute about 25% of *P. falciparum* malaria severity. However only small proportions (~ 2%) of heritability is explained by the well-known variants such as sickle-cell anaemia and α-thalassaemia [7]. The conventional approaches such as candidate gene-based studies [15–17] and the family based linkage studies [18, 19] have been implemented at least for the last three decades and identified several association variants. Unfortunately, the majority of the findings were discordant and failed to replicate in different populations [1].

GWAS in malaria susceptibility study was motivated to address the acute limitations of the conventional approaches and provide better understandings of the underpinning genetics at genome wide scale. To this effect, a global partnership of malaria researchers, named as Malaria Genomic Epidemiology Network (MalariaGEN) was established in 2008 [20]. MalariaGEN has successfully conducted multi-center-scale GWASs [10–13] and reported some interesting findings which we will discuss in later sections. However, the GWAS approach has several limitations including 1) weak performances in genetically diverse populations [8], lack of translation of associated loci into suitable biological hypotheses [21], 3) the well-known problem of missing heritability [22], 4) the lack of understanding of how multiple modestly associated loci within genes interact to influence a phenotype [23], 5) inefficiency in distinguishing between inflation from bias (cryptic relatedness and population stratification) and true signal from polygenicity [24], 6) the imperfection of asymptotic distribution of current mixed model association in the case of low-frequency variants [25]. The discussion on limitations of GWAS approache is beyond the scope of this review. Here we focus on the major challenges of malaria susceptibility GWASs and highlight the recent positive progresses.

#### Genetic diversity of African population

Owing to the fact that Africa is the origin of modern humans, there is high level of genetic diversity and weak linkage disequilibrium (LD) in Africans compared to non-African populations [26–28]. These distinct genetic characteristics created major setbacks to GWASs in African populations primarily because of lack of representative dense genotype chips and reference panels [8]. It was estimated that a GWAS of 0.6 million SNPs based on HapMap phase 1 dataset in European population has an equivalent power to the chips with 1.5 million SNPs in African populations [29].

This might have affected the power of previous malaria GWASs. For instance, in the first malaria GWAS [10], *HbS* locus, a well-known variant conferring resistance to severe malaria demonstrated a weak signal (p-values ~ 1x10^-7^) because of the weak LD between causal variants and the SNPs that were genotyped. After which authors sequenced the locus, undertook multipoint imputation, used proper reference panel and dramatically improved the signal to p-value ~1x10^-14^. However, coverages of the genotyping chips have been enormously improved to be able to capture the genetic diversities among global populations following the recent technological advances and availability of diverse reference data sets [27, 30–32]. For instance, Omni microarrays based GWASs were proven to have considerable power in African populations [27].

Such developments have also facilitated imputation-based studies in African populations. For instance, Band et al*.* [9] showed the feasibility of multi-point imputation based meta-analysis in for malaria GWASs using HapMap3 haplotype panel. Another study showed a substantial improvement of imputation accuracy by using the more diversified AGVP WGS reference panel [27]. We believe that the reference dataset will grow further and accelerate genomic research by including wide range of haplotype diversity in African populations.

#### Sample size

In GWAS, a stringent p-value (0.5x10^-8^) is usually needed to declare evidences of genuine associations to minimize false discovery rate that can arise from multiple testing [33]. Thus, very large sample size is required to achieve genome-wide significance threshold particularly for loci with modest effect sizes. The required sample size is even much higher for studies in population of African ancestry because of the higher genetic diversity. In contrast, the current sample sizes of GWASs in African populations including those of malaria susceptibility are generally small compared to non-Africans [34] which might have affected the power of the studies. Therefore, more powered studies in African population might lead to the discovery of novel association variants.

#### Allelic heterogeneity of malaria protective variants

Allelic heterogeneity defined as the presence of multiple causal variants in the same locus is one of the challenges of GWA and fine mapping studies [35]. The presence of multiple causal variants with variable effect sizes and LD structures limits the power of GWASs. In such cases, fine-mapping methods will also have lower accuracy to pinpoint true causal variants among several possible candidates [35]. Allelic heterogeneity has been described for the well-known loci affecting malaria susceptibility, which is reflected by their geographical distribution within malaria-endemic regions [36].

Several distinct variants are known to exist at the loci causing inherited hemoglobinopathies [36]. Allele frequencies, LD structure and effect sizes of these variants differ in sub-populations within endemic areas [37]. For instance, the sickle cell allele, *HbS*, is known to have different haplotype structure and effect sizes in different regions of sub-Saharan Africa [27]. *HbC* allele is common in some parts of west Africa such as Burkina Faso, Ghana, Togo and Benin while absent in other west African countries such as Cameroon and Chad [38]. In the same region, several alternative alleles with differing effect sizes are known to exist at the locus causing G6PD deficiency [36, 39]. Although population specific studies can minimize such challenges, the current MalariaGEN datasets are comprised of several populations each with small sample size; making it difficult to undertake powered GWASs for specific geographic areas.

#### Genetic architecture of malaria susceptibility and resistance

The performance of GWASs is dependent on the genetic architecture of the diseases and traits under investigation. For the majority of complex diseases and traits, the GWAS variants identified thus far, only explain a very small proportion of heritability; a phenomenon commonly termed as ‘missing’ heritability [22] . There have been different explanations for the ‘missing’ heritability including common disease rare variant hypothesis [40], none-additive components, primary epistasis [41, 42] and polygenic genetic architecture [21].

One of the challenges of malaria GWASs is that we don’t know much about the genetic architecture of malaria protection trait. First, as one of the prominent evolutionary selective forces, the majority of malaria protective alleles might have evolved under positive selection and might potentially be balanced by other forces [43]. In this case, the protective variants are expected to have large effect sizes with high allele frequencies that can be detected by the conventional GWAS approaches; provided that proper reference panel and genotyping platforms are used [43]. Second, similar to the genetic architecture of other infectious diseases [44], malaria protection trait might largely be attributed to few rare variants of large effect sizes. In this case, the GWASs are underpowered as rare variants might not be in LD with common variants. Third, malaria protection trait might be mainly under polygenic and epistatic control [1, 7, 45] which the conventional GWAS approach can’t capture.

### Biology of the novel variants identified by severe *P. falciparum* malaria GWASs

Severe malaria GWASs have replicated some of the well-known variants such as *HbS* and *ABO* blood groups and few novel variants related to red blood cell membrane biology which reinforce the importance of erythrocyte variants for protection against severe malaria. Besides, the GWASs have identified notable novel association variants in immune and other pathways that may directly or indirectly influence the disease outcome. The epidemiology and biology of the well-known variants were reviewed else-where [1]. Below we characterize the novel malaria susceptibility genetic variants identified by GWASs. We first discuss the biology of two variants such as cluster of the *glycophorin genes (GYPA/B/E)* and *ATP2B4* that were well-replicated across malaria endemic populations. We then extend our discussion to other novel variants.

#### ATP2B4

The association of variants in *ATP2B4* gene with severe malaria susceptibility was reported by Timmann et al. [11] in Ghanaian populations and replicated in subsequent studies in other populations [12, 46]. SNPs in this locus were also linked with reduction of mean corpuscular hemoglobin concentration (MCHC) level [47]. *ATP2B4* encodes a ubiquitous plasma membrane calcium-transporting protein (*PMCA4b)* [48]. *PMCA4b* is widely expressed in different tissues and is the main transporter of *Ca*^2+^ in erythrocyte membrane [48].

A recent study showed that the GWAS SNPs are localized in a previously unrecognized *ATP2B4* haplotype named as ‘haplotype-1' and individuals with this haplotype exhibit a reduced *PMCA4b* expression level [49]. In this study, it was also shown that the reduction of *PMCA4b* expression significantly decreases the calcium extrusion in RBCs. Consistent with this, a study conducted by Lessard et al*.* and colleagues elegantly characterized the *ATP2B4* locus using a combination of transcriptomic, epigenomic and gene-editing study approaches [50]. The authors first undertook knock out experiment and demonstrated that *ATP2B4* knocked-out mice express an elevated level of MCHC. Then, they conducted expression quantitative trait locus (eQTL) mapping studies using UK biobank dataset and showed strong associations between *ATP2B4* erythroblast specific variants and RBC related traits including MCHC level, decreased RBC distribution and increased hemoglobin levels.

Further analysis of DNase I hypersensitivity sites (DHSs) at *ATP2B4* and eQTL mapping showed that the GWAS SNPs are mapped to an erythroid specific enhancer element. Deletion of this enhancer from human erythroid cell line using CRISPR-Cas9 system showed a dose dependent reduction of *ATP2B4* expression level. Bi-allelic deletion of the enhancer resulted in eighty three percent reduction of *ATP2B4* expression level compared to the wild type while mono-allelic deletion resulted in moderate reduction of the *ATP2B4* expression level [50].

To determine the effects of the regulatory variants at *ATP2B4* gene on calcium homeostasis, Lessard et al*.* measured the calcium concentration in unedited and edited (*ATP2B4* enhancer deleted) HUDEP-2 cells. The edited cells demonstrated higher intracellular calcium level compared to wild cells indicating that *ATP2B4* expression is essential for plasma membrane calcium pump. The disturbance of intracellular *Ca*^2+^ homeostasis might play an important role in impairing the invasion, development and reproduction of malaria parasite in RBCs [51, 52]. Therefore, *ATP2B4* region can potentially be targeted for development of vaccine and therapeutics.

#### Cluster of the 3 glycophorin genes (GYPA/B/E)

The largest multi-center malaria susceptibility GWAS which included eleven populations was conducted by Band et al*.* [12]. In this study, 34 genomic regions containing potential susceptibility loci for severe malaria were identified. Among which, a strong signal was observed at locus between *FREM3* gene and cluster of 3 *glycophorin genes (GYPA/B/E*) on Chromosome 4. A haplotype (at SNP *rs184895969)* within this region was reported to reduce the risk of developing severe malaria by about 40% and is common in Kenyan populations with allele frequency reaching 10% [12].

A subsequent study in the same populations identified a large number of copy number variants which are characterized by deletion, duplications and hybrid structures in *GYPA* and *GYPB* genes [53]. Of which a distinct variant called *DUP4* was reported to reduce the risk of severe malaria by about 40% in eastern African (Kenya) populations. Further characterization showed that, this variant is composed of complex *GYPB-A* hybrid and encode *Dantu* antigen in MNS blood group system [53]. The association of this region with severe malaria was supported by another recent case control study in Tanzanian populations [54]. The glycophorin gene cluster, *GYPA* and *GYPB* encode the MNS blood group system and are known to be receptors for *P. falciparum* during RBC invasion [55]. *GYPA* and *GYPB* serve as an erythrocyte membrane receptor for *EBA-175* and *EBL-1* proteins of the parasite respectively [56]. This genomic region is also known to be under an ancient selective pressure resulted from host-pathogen arm races between *P.falciparum* and humans [57] . Further functional analysis is required to better understand how these variants affect the invasion and/or development of the parasites in erythrocytes and convey protection against severe malaria.

#### SCO1 and DDC

Notable association signals were identified by the first malaria susceptibility GWAS conducted in Gambian population [10]. The first lead SNP (*rs6503319*) is located close to *SCO1 (synthesis of cytochrome c oxidase)* gene on chromosome 17p13. *SCO1* is a multi-functional signaling protein which plays an essential role in *mitochondrial cytochrome c oxidase (COX)* copper delivery pathways [58]. *COX* catalyzes electron transfer from reduced cytochrome c to oxygen and is abundantly expressed in muscles, brain and liver [58]. Deficiency of *COX* caused by mutations in *SCO1* gene can lead to respiratory distress and severe metabolic acidosis [59] which are also the major complications during cerebral malaria [60]. Further studies are needed to understand how the variants in *SCO1* gene are associated with the pathological pathways of cerebral malaria.

The second notable association signal identified in this study was *Dihydroxypheny-alanine decarboxylase (DDC)* on Chromosome 7p12.2. A recent study in Tanzanian populations replicated the association of *DDC* variants with cerebral malaria [54]. *DDC* gene encodes *Aromatic-L-amino-acid decarboxlase* enzyme which is involved in biosynthesis of neurotransmitters such as dopamine and serotonin [61]. *DDC* is an essential enzyme for brain and nervous developments and its deficiency is associated with reduced cognitive functions [61]. *DDC* is involved in cellular immunity and contributes in protection against parasitic disease in invertebrates [62]. Furthermore, mutations in *DDC* gene was reported to be associated with refractoriness of *Anopheles gambiae* mosquito against *P.falciparum* parasites [63]

#### MARVELD3

In addition to the *ATP2B4*, Timmann et al*.* [11] identified an association SNP (*rs2334880*) on chromosome 16p 22.2 which is linked to *MARVELD3*. However, this association has not been replicated in other studies. *MARVELD3* is one of the components of tight junction proteins in several epithelial and endothelial tissues and is expressed as two alternative spliced variants [64]. These proteins are involved in assembly, development, maintenances and regulations of tight junction. Tight junctions play a major role in intracellular adhesions and involved in sub-cellular signaling mechanisms [64].

#### IL-12 receptors and IL-23 receptors

The most recent malaria susceptibility GWAS was conducted in Tanzanian population [13]. In this study, notable associations signals were identified in immune pathways including in interleukin receptors (*IL-23R and IL-12RBR2*), *in ketch-like proteins (KLHL3)* and Human Leucocyte Antigen (HLA) regions. *Interleukin-12* is formed from a hetrodimer of *IL12B (ILp40 subunit)* and *IL-12A (ILp35 subunit*) [65]. *IL-12* plays a vital role in stimulating cell-mediated immune responses against intra-cellular pathogens through binding to high affinity *IL-12RB1* and *IL-12RBR2* receptor complexes. It promotes the development of *T-helper cells* (*Th1)* and enhances the production of *INF-γ*, both of which are known to mediate the clearance of intracellular pathogens [65]. In malaria, *IL-12* has been implicated in mediating the protective immunity both in experimental animals and in humans [66] . *IL-23* is an important pro-inflammatory cytokine that shares p40 subunits with *IL12* [67]. It induces the differentiation of naive *CD4 T*-cells to *IL-17* which plays key roles in pathogenesis of autoimmune diseases [68].

HLA is encoded by the Major Histocompatibility Complex (MHC), the most polymorphic genes known in human genome. The diversity of MHC is believed to be driven by selection pressure from infectious pathogens and known to be associated with the risk of several infectious diseases [69]. HLA variants such as HLA class I antigen (*HLA-Bw53*) and HLA class II variant (DRB1*1302-DQB1*0501) were reported to confer protections against severe malaria in Gambian populations [69]. HLA class I antigen is expressed by liver cells suggesting that T cells (CTL) responses might efficiently act against the liver stage of malaria parasite in individuals with *HLA-Bw53* [69]. On the other hand, individuals with DRB1*1302-DQB1*0501 variant might possess efficient antigen presentation mechanism that can lead to rapid clearance of blood stage parasites [69].

Variants in immune pathways are of great interest because of their potential to inform the development of effective malaria vaccines [1]. The current study is interesting in that several putative variants in immune pathways were identified. However, the power of this study is limited because of relatively smaller sample size and weak significance threshold used to interpret the findings. Therefore, further studies with higher detective power are needed to consolidate the findings (Table 1).

**Table 1:** Summary of the novel severe malaria susceptibility and resistance association variants identified by GWASs

Genomic regions containing the association variants					Genome-wide association studies							
					Jallow *et.al* [10] Pop: Gambian N = 2560(case =1060, control = 1500)		Timmann et al. [11] Pop: Ghanaian N = 2153 (case =1325, control =828)		Band et al. [12] Pop: African (11 countries) N = 11,552 (case = 5633, control = 5919)		Ravenhall et al. [13] Pop: Tanzanian N = 914 case =449, control = 465)	
Nearest gene name	Chr	Position	SNP ID (Ref/Alt)	MOI	OR	*p*-value	OR	*P*-value	OR	*P*-value	OR	*P*-value
*ATP2B4*	01	203658471	rs 4951377 (A/G)	DO	–	–	–	–	–	3.1x10^−9^	–	–
		203654024	rs 10900585(T/G)	AD	–	–	0.61	1.9 × 10^−10^	–	–	–	–
		203660781	rs4951074(G/A)	AD	–	–	0.62	1.3 × 10^−9^	–	–	–	–
*IL23R, IL12RB*	01	67,731,614	rs6682413(−)	RE	–	–	–	–	–	–	0.48	8 × 10^−7^
*GYP A/B/E and FREM3*	04	143777125	rs184895969(A/C)	DO	–	–	–	–	0.67	9.5 × 10^−11^	-	-
*C4orf17*	04	100429757	rs73832816(−)	REC	–	–	–	–	–	–	0.29	3.8 × 10^− 7^
AF146191.4–004 (lincRNA)	04	90717704	rs114169033(−)	AD	-	-	-	-	-	-	3.32	6.7 × 10^−7^
*AC108142.1* (antisense)	04	82822332	rs1878468	HET	-	-	-	-	-	-	0.383	9.0 × 10^−7^
*Intergenic*	05	43,909,343	rs113449872(−)	HET	–	–		–	–	–	0.35	2.2 × 10^−8^
*KLHL3, MYOT*	05	37,011,761	rs2967790(−)	AD	–	–	–	–	–	–	0.60	5.9 × 10^−7^
*TREML4*	06	41,205,690	rs9296359 (−)	HET	–	–	–	–	–	–	4.08	1.2 × 10^−7^
*DDC*	07	50,623,201	rs10249420(C/G)	AD	0.69	6.8 × 10^−5^	–	–	–	–	–	–
			rs1451375(−)	DO	0.75	6.1x10^−6^	–	–	–	–	–	–
*Intergenic*	07	53,676,837	rs17624383(−)	AD	–	–	–	–	–	–	–	5.6 × 10^−7^
*CSMD1*	08	4754838	rs73505850(−)	AD		–	–	–	–	–	4.79	5.9 × 10^− 7^
*LINC00944*	12	127237620	rs11335470 (−)	HET	–	–	–	–	–	–	0.40	2.5 × 10^−7^
*Intergenic*	11	130,417,522	rs3133394	AD							0.5	9.4X10^−7^
*FAM155A*	13	108228013	rs144312179(−)	AD	–	–	–	–	–	–	0.2	6.2 × 10^−7^
*MARVELD3*	16	71,653,637	rs2334880 (T/C)	AD	–	–	1.19	1.9 × 10^−6^	–	–	–	–
*SOC1*	17	10,573,909	rs65033119(−)	AD	1.21	7.2 × 10^−7^	–	–	–	–	–	–
*Intergenic (LINC00670)*	17	12,399,526	rs149085856(−)	AD	–	–	–	–	–	–	3.87	2.1x10^−7^
*ZNF536*	19	1,069,639	rs8109875(−)	REC	–	–	–	–	–	–	0.5	5.7 × 10^−7^

*MIO* Mode of inheritance, *AD* Additive, *HET* Heterozygous, *DO* Dominant, *REC* Recessive, *OR* Odd-ratio, *Ref* Reference allele, *Alt* Alternative allele, *Pop* Population

### Polygenic genetic architecture and epistasis: Presenting the absent in the current severe malaria GWASs

#### Polygenic genetic architecture

Polygenic view of genetic architecture is gaining ground in genetic epidemiological studies and widely implicated for the ‘missing’ of heritability in GWAS analysis [70] . The rationale behind polygenic inheritance is that complex-traits/diseases are influenced by multiple variants with modest effects that are too small to pass the stringent genome wide significance threshold [71]. In standard GWAS analysis, ‘Genomic control’ (GC) method is applied as a quality-control measure to minimize spurious associations that can be caused by population structures such as population stratification and cryptic relatedness.

However, a slight inflation of the test statistics (true but weak signals) which cannot be corrected by GC was initially observed across the genome in a Schizophrenia GWAS [72]. Subsequently, this observation has been supported by other studies [73] and led to the development of a number of statistical tools aiming to capture polygenic signals at genome-wide scale including 1) polygenic scoring method implemented in PLINK software [74] ; 2) Mixed Linear Models (MLM) such as: GCTA [73], BOLT-LMM [75] , Bayes-R [76] and LDAK [77, 78], PCGC [79] 3) linkage-disequilibrium (LD) score regression method [80] among others.

#### Polygenic contributions in malaria susceptibility and resistance

Co-evolution of host-pathogen model predicts that multiple host loci are involved in resistance/susceptibility to infectious diseases due to the complex interactions between the multi-locus parasite genotype and the corresponding defense from the host-genome [42, 81]. Indeed, malaria might have left multiple genetic variants; the majority of which have effects too small to be detected by the standard GWASs. The existence of polygenic inheritance in malaria protection was predicted by several authors [1, 7] and supported by the GWASs. For instance, the largest malaria susceptibility GWAS so far, identified 34 regions of the genome containing variants with evidence of associations [12]. Earlier GWAS in Ghanaian population identified 40 genomic regions containing 102 SNPs with evidences of association in the discovery phase of the study [11]. The recent GWAS in Tanzanian populations [13] identified 2322 SNPs at several regions across the genome.

Thus, implementation of polygenic analytic methods in malaria studies may potentially shed more light to the underlying biology. For instance, heritability can be estimated and partitioned in to different cell-types and functional groups and molecular pathways which enable to localize causal variants. Furthermore, these approaches can be extended to explore the genetic correlations between susceptibility to malaria and  susceptibility to other infectious diseases. The existence of shared genetic basis between infectious diseases susceptibility/protection is well-documented [82]. However, the extent and pattern of these correlations have not been systematically investigated at genome-wide scale; partly because of inadequate GWAS data for infectious diseases. Such studies can potentially provide clues to common molecular processes between resistance/susceptibility to infectious diseases that will have practical importance including designing multi-purpose vaccine and genetic risk prediction strategies.

#### Heritability of severe malaria in Gambian population

To figure out how polygenic analysis can be implemented in malaria susceptibility, we accessed the Gambian malaria susceptibility GWAS dataset from European Phenome Genome Archive (EGA) through data application procedure and estimated heritability of malaria susceptibility using MLM approaches. The Gambian GWAS data is the largest MalariaGen dataset obtained from a single country comprised of 4920 samples (2429 cases and 2491controls) and 1.6 million SNPs that passed GWAS quality control (QC). We first excluded the known malaria susceptibility associated loci and performed stepwise extra QC filtering. Specifically, we focused on sample relatedness, SNP missingness proportion and SNP differential missing proportion which are well-known to affect the accuracy of heritability estimation [78]. We then estimated the heritability using GCTA model for different QC thresholds by including 10 principal components (PCs) as fixed effects to account for population structure. As expected, the estimation was unstable when less stringent QC thresholds is applied (varying from 37.8 to 20.1%) as shown in Table 2. However, when more stringent QC (Relatedness threshold (5%), SNP differential missingness proportion (p < 1 × 10 ^− 3^) and SNPs missing proportion of (p > 0.02)) was  applied, the estimation became stable (~ 20.1%, SE = .05). Neither the inclusion of more PCs (15, 20) as fixed effects nor SNP phasing further brought down the estimate. Using the same stringent QC threshold, the estimation was approximately the same for Mandinka ethnic group (~ 24.3%, SE = 0.6). We couldn’t estimate for other ethnic groups because of smaller sample sizes. Furthermore, the use of PCGC model which is designed for case/control approximately showed the same estimate (19.8%, SE = .07).

**Table 2:** SNP-heritability of severe malaria susceptibility/resistance in Gambian population at different basic quality threshold using MLM

Population	Sample relatedness-threshold	SNP missingness-proportion	SNP differential-missingness Proportion	Prevalence	Covariate principal-components	No. Samples	No. SNPs	GCTA h2(% SE)	PCGC h2( SE)
Gambia	–	5%	–	1%	10	4920	1627656	37.8(.05).	
	5%	5%	1 × 10^−10^	1%	10	4128	1627656	30.5(.05)	
	5%	5%	1x10^− 5^	1%	10	4128	1607610	28.7(.05)	
	5%	5%	1 × 10^−3^	1%	10	4128	1570344	25.1(.05)	
	5%	2%	1 × 10^− 3^	1%	10	4128	1486554	20.1(.05)	19.8(.07)
	5%	2%	1 × 10^−3^	1%	15	4128	1486554	22.5(.05)	
	5%	2%	1x10^−3^	1%	20	4128	1486554	19.5(.05)	
	Phased	–	–	1%	10	4128	1627656	20.4(.06)	
Mandinka	5%	2%	1x10^−3^	1%	10	1281	1486554	24.2(0.6)	

*GCTA* Genome Complex Trait Analysis, *PCGC* Phenotype Correlation Genotype Correlation regression

Although our heritability estimation is fairly stable when stringent QC is implemented, care should be taken in interpreting these results: First, all polygenic methods perform better in less structured data obtained from homogenous populations than the MalariaGen dataset which is comprised of diverse populations spanning most of the Malaria endemic belt in Africa. Second, the methods are designed and perform well in highly polygenic traits/diseases in which effects of each variant is mostly modest. In contrast, a considerable proportion of malaria protection trait might be attributed to rare variants of large effect sizes that might not be in LD with common variants and can’t not be ‘tagged’ by SNPs chip which means that the contributions of such variants will not be accounted for. Third, subtle population structure that cannot be corrected by conventional methods such as unmatched case/control can potentially create systematic biases to the estimates.

#### Epistasis

Epistasis is becoming one of the hot research topics in genetic epidemiological studies in the last few years because of the fact that none additive genetic variations are shown to have significant influence on the phenotype of complex traits/diseases than previously expected [83]. The available statistical approaches and software packages for detection of epistasis at genome-wide scale were reviewed elsewhere [83, 84]. These approaches have been applied in genetic studies of complex diseases such as lupus erythematosus [85], anklosing spondlylitis [86], psiorosis [87] and unraveled previously unknown epistasis interactions between risk loci which explained a significant proportion of ‘missing’ heritability of the respective diseases.

Epistasis between malaria risk loci have been well documented and implicated as one of the possible reasons for lack of replication of susceptibility variants in different populations and the ‘missing’ heritability. For instance, sickle-cell trait (*HbS*) and *α thalassaemia* were shown to demonstrate negative epistatic interactions such that the protection against severe malaria offered by *HbS* is reduced when co-inherited with *α*^*+*^ *thalassaemia* [88].

A case-control study in Kenyan population also reported that *α*^*+*^ *thalassaemia* modulates the effects of Haptoglobin (Hp) variants in predicting the risk of severe malaria [89]. In this study, it was shown that the combination of *α*^+^ *thalassaemia* and *Hp2–1* variant synergistically increase the protection against severe malaria by about 37%. However, the protective effect is decreased to 13% when *α* ^+^ *thalassaemia* is inherited with *Hp1–1* and further diminished to neutral (zero) when inherited with *Hp2–2*. Similarly, in a multi-center case control study, the existence of negative epistasis interaction between *HbC* and *ATP2B* alleles was reported [15].

Another recent case control study of severe malaria in Kenya reported the existence of negative epistasis between a compliment receptor called *S12* and *α*^*+*^*thalassaemia* in which the protective effect of *S12* higher in children with normal *α-globin* [90]. The extent and pattern of epistatic interaction at genome-wide scale is yet to be explored. In malaria susceptibility GWAS, the priority has been given to a single locus analysis to identify novel risk loci. We expect that, the next step of malaria susceptibility GWAS will include the investigations of epistasis at genome-wide scale.

### From GWAS to biology: multi-step and multi-dimensional analyses

#### Fine-mapping and pathway analyses

The ultimate goal of genetic susceptibility studies is to identify causal variants and understand the underlying biological pathways which can lead to translated medicine such as effective vaccines and therapeutics. However, translating GWAS signals in to biological themes remains an open problem because of the confounding effects from LD between association SNPs, limited knowledge of gene functions and localization of the majority of GWASs hits in none protein coding regulatory regions (regulatory SNPs) [91–93]. In attempt to address this challenge, several fine mapping strategies have been developed and implemented [94]. One such strategy is trans-ethnic fine mapping in which the natural variability of haplotype structure across ethnically diverse populations is used to narrow down candidate causative variants [95]. The smaller LD and diversity of haplotype structure in African population makes it relatively easier to identify the causal SNPs and target gene/genes through fine mapping approaches [8]. However, the fact that malaria protective alleles are heterogeneous across populations might challenge the application of trans-ethnic fine-mapping approaches in malaria susceptibility studies.

Alternatively, several fine mapping statistical tools have recently been developed following the advances in annotation data bases and improved reference panels. These include Bayesian approach, heuristic approach and penalized regression methods [96]. The principles, applications, strength and weakness of these methods is reviewed elsewhere [94, 96]. These methods are increasingly playing crucial role in the efforts being made to pinpoint causal variants of complex diseases/traits. For instance, Galarneau et al. [97] identified novel independent association signals by fine-mapping three loci that are known to influence fetal hemoglobin (HbF) levels. The authors sequenced the three loci (*BCL11A, β-globin and HBS1L-MYB*), undertook dense genotyping, performed step-wise conditional analysis and revealed previously un-recognized SNPs that explain additional genetic variation. Similarly, a recent fine mapping study of HLA region identified several susceptibility loci for multiple infectious diseases [82]. More sophisticated studies that combine statistical and functional fine-mapping strategies have recently been implemented and provided mechanistic insights to the genetic basis of complex diseases [98].

In addition to fine mapping approaches, pathway and interaction analysis can be another avenue for exploring molecular basis of Malaria susceptibility/resistance. Instead of emphasizing on single-variant analysis, these approaches test the coordinated effects of several variants at systems level using biological information from annotation data basis [99]. Pathway analysis improve study power by integrating cumulative effects of weak association signals and provide functional information by identifying associated sets of genes/proteins [100]. By implementing the pathway analysis approaches, several studies have gained new insights in understanding the genetic basis of complex diseases [101–103]. The available statistical tools for pathway and interaction analysis is reviewed in [104]. We therefore, advocate for the implementation of fine mapping and pathway analytic methods in malaria susceptibility studies to shed more light in to the underlying biology.

#### Epigenomics and Epigenome wide association studies

Epigenetics refers to heritable phenotype changes that do not involve alterations in the DNA sequences such as methylation, post-translational histone modification, histone variation, chromatin remodelling and non-coding RNAs [105]. Epigenetic impacts have recently been implicated in malaria susceptibility and resistance [106, 107]. For instance, in a recent study, strong transcriptional response was detected in monocytes of *P. falciparum* infected individuals from Fulani, an ethnic group that is less susceptible to malaria [107]. The authors suggested that, this response is likely regulated by genome wide chromatin alterations. The discussion on the possible mechanisms of epigenetic impacts on malaria susceptibility and resistance is beyond the scope of this paper and is reviewed in [108].

However, the majorities of epigenetic studies including those of malaria susceptibility and resistance have been limited to either small sample sizes or inadequate genome coverage and thus, lack adequate power to decipher the epigenetic impacts on complex diseases [105]. In effort to address this challenge, investigators recently developed a large-scale, systematic epigenomic equivalents of GWAS called epigenome-wide association studies (EWAS) that attempts to uncover epigenetic variants underlying common diseases/phenotype using genome-wide technologies such as Illumina 450 K array [109]. EWA approach recently gains a considerable attention partly due to the fact that the majorities of the GWAS SNPs are mapped to none coding regions of the genome implying that the variant SNPs cause changes in gene expression levels rather than causing changes in protein function [109]. Thus, combining both genetic (GWA) and epigenetic (EWA) approaches in parallel may prove a fruitful approach for understanding mechanisms of disease risk [109]. Undoubtedly, application of such approaches may shed new light into mechanisms of malaria susceptibility and protection.

#### Multi-omics approaches

Today, there are significant advances in high throughput technologies that can generate big ‘-omic’ data from all spectrum of molecular biology [91]. The ‘-omics’ studies (Genomics, Epigenomics, Transcriptomics, Proteomics, Metabolomics) are crucial to understand the underpinning biology of complex diseases. In severe malaria, ‘-omics’ studies have provided important clues about the molecular events that lead to either complications or recovery from diseases. For instance, following the discovery of glycophorin regions by the GWAS, a whole genome sequencing-based study [53] was conducted to characterize variants in this region and identified a novel distinct copy number variant called *DUP4* which reduces the risk of severe malaria by about 33% . In addition to this, a genome-wide gene expression study was conducted in Kenyan children and reported increased expression of genes related to neutrophil activation during malaria infections [110]. The authors also observed differential expression of heme- and erythrocytes-related genes in acute malaria patients which reaffirms the importance of erythrocyte-related genes in malaria susceptibility and resistance.

In another host-parasite interaction study, the importance of miRNA in inhibiting parasite growth in erythrocyte was reported [111]. The authors observed translocation of several host RBC miRNAs in to *P. falciparum* parasites, as well as fusion of these human miRNAs with parasite mRNA transcripts to inhibit the translation of enzymes that are vital for the parasite development. Specifically, two micro-RNAs, miRNA-451 and let-7i, were highly enriched in *HbAS* and *HbSS* erythrocytes and these miRNAS along with miR-223 were shown to attenuate the growth of parasite [111].

However, ‘-omics’ studies are limited to single data-type analysis and lack adequate power to explain the complexity of molecular processes and usually lead to identification of correlations than causations [112]. Thus, integrating and analysing multiple ‘-omics’ data enables better understanding of the molecular processes and interactions that give rise to complex diseases/traits. For example, leveraging host microbiome relative abundance data as a second (quantitative) trait, and performing a joint analysis of bivariate phenotypes can increase statistical power by maximizing phenotypic information and inform how the interaction between host genotype with microbiome impacts the phenotype.

Multi-omics approaches aim to integrate big ‘-omics’ data, undertake ‘multi-step’ and ‘multidimensional’ analysis for elucidating complex biological problems [112] . Driven by the massive abundance of ‘-omics’ data from wide ranges of biological molecules, multi-omics strategy have recently provided unprecedented successes in complex diseases/trait studies. The current state of art of multi-omics approach and available statistical methods is recently reviewed in Hasin et al*.* [112]

**Fig. 1:**
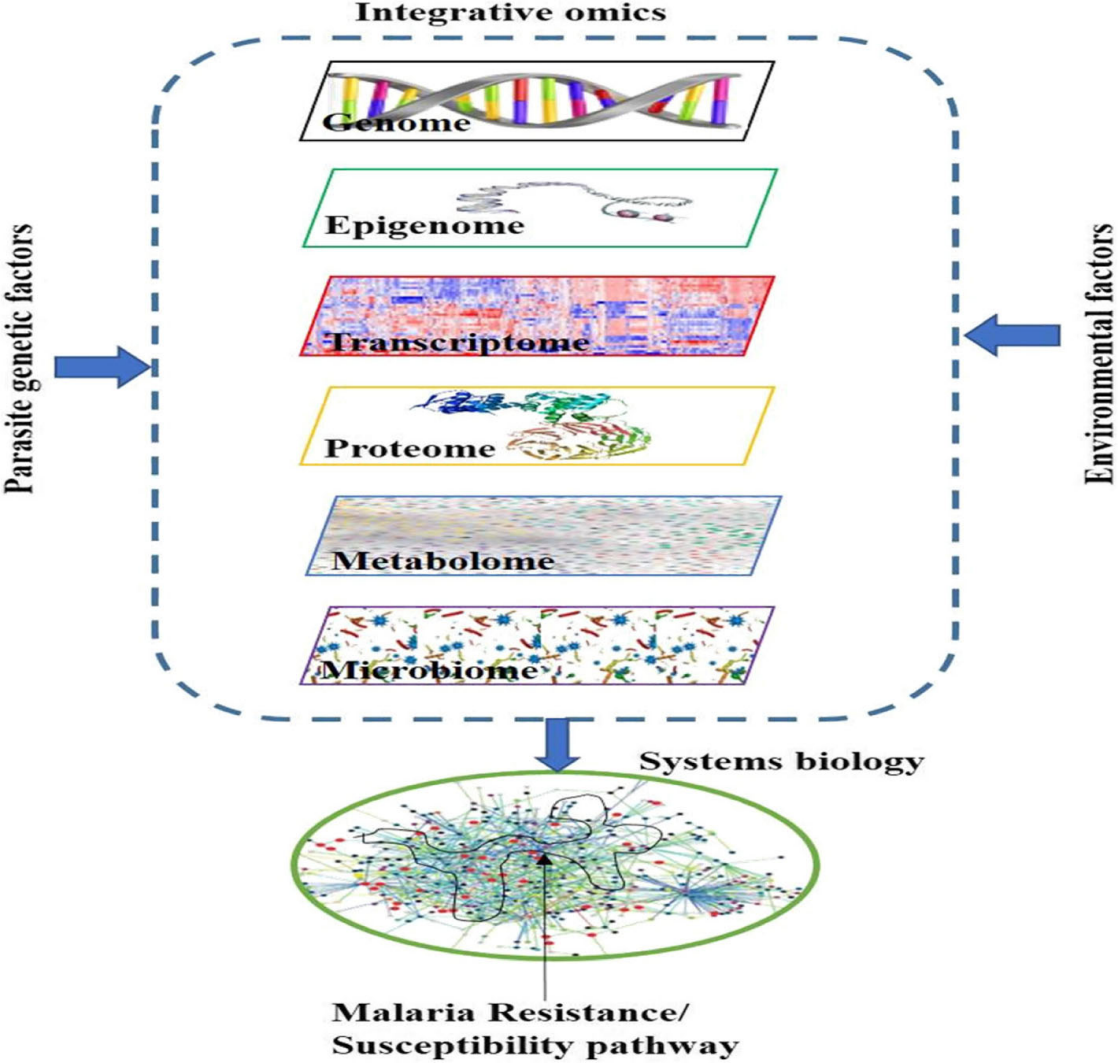
Schematic representation of the integrative analyses. Systems biology approach which incorporate multiple layers of information from host (multi-omics), the environment and parasite genetic factors can potentially lead to the discovery of malaria protective pathways

Malaria susceptibility and resistance is influenced by several host, parasite and environmental factors as depicted in Fig. 1. The protective alleles have independently evolved in different populations being shaped by the co-evolution and interaction between the human genome, the parasite and the environment [1]. Thus far, single ‘-omics’ data analysis enabled us to understand some of the factors that are associated with the malaria protective traits. To progress beyond associations and pinpoint the causal pathways, it may require to implement carefully designed, coordinated multi-omics studies that involve human host, the parasite, the environment and possibly mosquito. The current advents of high-throughput technologies in generating massive ‘-omics’ data and their continuously decreasing cost complemented with the availability of statistical tools which able to simultaneously capture millions of data points will lead to the implementations of multi-omics approaches in malaria susceptibility studies.

## Conclusions and perspectives

The ultimate goal of malaria susceptibility study is to discover a novel causal biological pathway that provide protections against severe malaria; a fundamental step towards translational medicine such as development of vaccine and new therapeutics that can facilitate the global malaria eradication efforts. To achieve this goal, various study approaches have been implemented at least for the last three decades and successfully identified several association variants.

Recently, a number of GWASs have been implemented in malaria endemic areas to better understand the underlying biology. While some of the well-known variants were replicated, only few novel variants were convincingly identified and their biological functions remains to be understood. Several limiting factors including genetic diversity of population in malaria endemic areas, allelic heterogeneity of protective variants, small sample sizes, lack of proper reference panel and proper genotyping chips might have negatively impacted the malaria GWASs.

Another challenge is that we don’t know much about the genetic architecture of malaria protective trait. There are at least two scenarios in which GWAS approach might fail; First, malaria protective trait might largely be attributed to rare variants of large effect sizes that might not be in LD with common variants and can’t be captured by the GWAS approach. Second, malaria might have left multiple genetic variants distributed across human genome; the majority of which have effects too small to be detected by the standard GWASs [1, 7]. Theoretically, the large sample sizes, dense genotyping chips or whole genome sequencing, use of appropriate reference panels and effective genotype imputation can address the majority of the challenges. However, given the resource constraints; especially, in Africa where malaria problem is the greatest, this will likely take several years to achieve.

On the other hand, the recent advances in statistical techniques is enabling to extract useful information from the present-day GWAS sample sizes. For example, a number of statistical approaches have been developed to capture polygenicity in complex diseases. We showed how these methods can potentially be implemented in malaria susceptibility studies and provide useful insights. We believe that further studies with larger sample sizes can elucidate the polygenic effects in malaria protective trait by extending the analysis to genome partitioning, risk prediction and genetic correlations.

Beyond singe locus analysis, multi-step and multi-locus analyses including pathway analysis, fine mapping and interaction analysis can potentially be implemented in malaria susceptibility GWASs to gain new insights to the underpinning biology. For instance, pathway analysis can provide important information by analyzing the coordinated effects of several variants at systems level using biological information from annotation databases. Methods that combine statistical and functional fine-mapping strategies can potentially be implemented to pinpoint the causal variants from the GWAS association signals.

Most importantly, the future direction of malaria susceptibility requires a paradigm shift from single ‘-omics’ to multi-stage and multi-dimensional integrative functional studies that combines multiple data types from the human host, the parasite, the mosquitoes and the environment. The current biotechnological advances, an ever-increasing annotation data bases and availability of advanced analytical techniques, will eventually lead to feasibility of systems biology studies and revolutionize malaria research.

## Data Availability

Not applicable.

## Abbreviations

eQTL: Expression quantitative trait locus
EWAS: Epigenome wide association studies
GWAS: Genome wide association study
MLM: Mixed Linear Model
